# Prevalence and Antimicrobial Susceptibility of Bacterial Uropathogens Isolated from Pediatric Patients at Yekatit 12 Hospital Medical College, Addis Ababa, Ethiopia

**DOI:** 10.1155/2018/8492309

**Published:** 2018-10-02

**Authors:** Yamirot Merga Duffa, Kumera Terfa Kitila, Dereje Mamuye Gebretsadik, Adane Bitew

**Affiliations:** ^1^Yekatit 12 Hospital Medical College, Departments of Microbiology, Addis Ababa, Ethiopia; ^2^Addis Ababa University, College of Health Sciences, Department of Medical Laboratory Sciences, Addis Ababa, Ethiopia; ^3^Ethiopia Public Health Institute (EPHI), Addis Ababa, Ethiopia

## Abstract

**Background:**

Urinary tract infection (UTI) is considered as the most common bacterial infection seen among the pediatric patients.

**Objective:**

This study was carried out in order to determine the prevalence of urinary tract infection in pediatric patients, identify bacterial uropathogens responsible for the infection, and study the antibiotic sensitivity patterns of bacterial isolates.

**Materials and Methods:**

A cross-sectional study designed and conducted from January to April 2014. Clean-voided midstream urine specimens were obtained from 384 pediatric patients less than or equal to 15 years in sterile universal bottles. Urine collected from each patient was inoculated onto CLED and blood agar plates using a calibrated inoculating loop with a capacity of 0.001 ml. Inoculated plates were incubated for 24–48 hours at 37°C at inverted position aerobically. Bacterial isolates were indentified and characterized by Gram stain and by using an array of standard routine biochemical tests. The antimicrobial susceptibility test was carried out by using the Kirby–Bauer disc diffusion method. Frequency distribution tables were used to describe the findings. Logistical regression was also used to estimate crude odds ratio (COR) with 95% confidence interval (CI) of positive responses to the different variables, and *P* values less than 0.05 were taken as statistically significant.

**Result:**

In this study, a total of 384 patients (199 males and 185 females) aged less than or equal to 15 years from whom urine samples were collected were enrolled. Of these patients, 61 (15.9%) had significant bacteriuria. Of the 185 females, 36 (19.5%) came up with positive cultures, while 25 (12.6%) of the 199 males had significant bacteriuria, and the largest number of study subjects were below the age of 3 years, and the largest positive culture was obtained from this age group, accounting for 35 (57.4%.) out of 61 positive cultures. Bacterial species belonging to six genera were isolated and identified from 61 positive cultures, and the genera were *Escherichia*, *Klebsiella*, *Staphylococcus*, *Proteus*, *Acinetobacter*, and *Enterococcus. E. coli* was isolated in 28 cases (49.5 %), followed by *Klebsiella* spp. in 17 cases (27.9%), *Staphylococcus* spp. in 5 patients (8.2%.) (*S. aureus* in one and coagulase-negative staphylococci in 4 cases), *Enterococcus* in 7 cases (11.5%), *Proteus* spp. in 3 cases (4.9%), and *Acinetobacter* in one case (1.6%). Of the bacterial isolates, *E. coli* was found out to be the most common pathogen followed by *Klebsiella* spp. Furthermore, *E. coli* and *Klebsiella* spp. were the most common pathogens in female patients accounting for 71.4% and 64.7%, respectively. Regarding susceptibility tests, *E. coli* and *Klebsiella* spp. were not 100% susceptible to any of the 11 antibiotics tested. *Acinetobacter* spp. had 100% resistance to three antibiotics: gentamicin (GN), trimethoprim-sulfamethoxazole (SXM), and augmentin (AMP). But they were 100% susceptible to ciprofloxacin (CIP), cefuroxime (CXM), norfloxacin (NOR), and ceftazidime (CAZ). On the contrary, *Proteus* spp. was 100% sensitive to all drugs except to nitrofurantoin. Species of *Enterococcus* had resistance of 71.4% to chloramphenicol (C) and 85.7% to both SXM and erythromycin. *S. aureus* was 100% susceptible to almost all drugs, while coagulase-negative staphylococci were not as susceptible as *S. aureus*. Multidrug resistance to two or more drugs was observed in 73.7% of the bacterial isolates.

**Conclusion:**

This study determined the prevalence of urinary tract infection in pediatric patients and highlighted the major bacterial uropathogens involved in UTI for the first time in the country. Furthermore, bacterial pathogen species and their frequency was consistent with the usually reported pattern, with *E. coli* being the most common organism isolated in cases of urinary tract infections followed by *Klebsiella* spp. Most of the bacterial isolates were multidrug resistant, and it is therefore suggested that appropriate antimicrobials should be administered to reduce the risk of multidrug resistant organisms developing and avert ineffectiveness of antibiotics. This condition indicates that antibiotic selection should be based on knowledge of the local prevalence of bacterial organisms and antibiotic sensitivities rather than empirical treatment. The present study indicated that ciprofloxacin (CIP), ceftazidime (CAZ), cefotaxime (CTX), cefuroxime (CXM), clindamycin (DA), and ceftriaxone (CRO) were the best antibiotics for the treatment of Gram-negative and Gram-positive bacterial uropathogens, respectively, in the study area relatively.

## 1. Background

Urinary tract infections (UTIs) are a common and important clinical problem in childhood and may lead to systemic illness and renal injury in the short term, and repeated infections, renal scarring, hypertension, and end-stage renal dysfunction may develop [1]. It is a serious health problem, and it has been estimated that about six million patients visit outpatient departments and about 300,000 are treated in the wards every year for UTI worldwide [2]. UTI is also a common and important clinical problem in pediatrics, as recurrent UTIs may lead to renal scarring, hypertension, and end-stage renal dysfunction later in life [3]. UTI occurs in 3% to 5% of girls and 1% of boys during childhood, with the first attack occurring in girls by 5 years, peaking during infancy and toilet training, while it is common in boys during the first year of life, especially among those who are uncircumcised [4]. UTI is caused mainly by bacteria, although viruses, fungi, and parasites can also cause urinary tract infections. Among bacteria, Gram-negative organisms are most commonly isolated from urine samples of children with *Escherichia coli* (*E. coli*) accounting for 70% to 90% of infections [5]. *Klebsiella* species, *Proteus mirabilis*, *Pseudomonas aeruginosa*, *Acinetobacter*, and *Serratia* are other Gram-negative bacteria isolated from pediatric patients. However, only 10% of the cases are caused by Gram-positive bacteria and include *Enterococcus*, *Staphylococcus*, and *Streptococcus agalactiae* [6].

In recent years, widespread use of antibiotics has resulted in an increasing incidence of antibiotic resistance among the urinary tract pathogens all over the world. Worldwide, emergence of antibiotic resistance is increasing among the urinary pathogens [7]. More than 80% of bacterial strains causing urinary tract infections in developing countries are now resistant to trimethoprim or trimethoprim-sulfamethoxazole [8].

Urinary tract infection (UTI) is one of the most common bacterial infections encountered by clinicians in developing countries and the cause of significant morbidity and mortality [9]. Several studies from the African continent have investigated the profile of common uropathogens and the pattern of their susceptibility to commonly used antimicrobial agents in order to guide the choice of empiric therapy. These studies reported the emergence of antibiotic-resistant Gram-negative bacilli with special emphasis on ESBL-producing isolates [10–12].

However, to the best of our knowledge, no such studies have been published describing the spectrum of uropathogens in pediatric patients and their antimicrobial susceptibility patterns in Ethiopia.

Therefore, this research was conducted with the aim of investigating the prevalence and patterns of antibiotic resistance of uropathogens among pediatric patients less than or equal to fifteen years at Yekatit 12 Teaching Medical Hospital, Addis Ababa, Ethiopia.

## 2. Materials and Methods

### 2.1. Study Area

The study was conducted at Yekatit 12 Hospital Medical College, which is located in the center of Addis Ababa. Yekatit 12 Teaching Hospital is one of the hospitals under Addis Ababa City Administration Health Bureau that has been giving routine health services for the city community and other referral cases from different regional states of Ethiopia. It is a tertiary level referral and teaching hospital in Addis Ababa that serves for a large number of people from the surrounding zones and nearby regions both for inpatient and outpatient treatment. This teaching hospital consists of an operating room, intensive care unit (ICU), and pediatric department with 12 beds, 13 wards with 327 beds, and outpatient departments.

### 2.2. Study Design and Period

It was a cross-sectional study designed and conducted with the objective of isolating, characterizing uropathogens in pediatric patients, and determining the antimicrobial susceptibility pattern of the isolates. The study was conducted prospectively from January to April 2014.

### 2.3. Source of Population

All pediatric patients not greater than 15 years of age attending Yekatit 12 Hospital Medical College were the source of population.

### 2.4. Study Population

All pediatric patients less than or equal to 15 years of age who were sent to the laboratory for urinalysis were included in the study.

### 2.5. Inclusion and Exclusion Criteria

Pediatric patients less than or equal to 15 years of age with urinalysis were included in the study, whereas pediatric patients who were taking antibiotics and mothers/guardians of pediatrics who did not volunteer for written consents were excluded.

### 2.6. Sample Size Determination and Sampling Technique

Since data were not available on pediatric bacterial UTI in Ethiopia, 50% of population proportion was used to determine the sample size based on single population proportion, and the level of precision (d) was 0.05. Urine specimens were collected from each pediatric patient. The study participants' parents or guardians were given appropriate instructions of clean catch midstream urine sample collection before providing urine samples, and immediately after collection, the samples were brought to microbiology laboratory for further process. Sterile plastic bags were used for admitted pediatric patients, and the bags were checked every 15 minutes for urine samples. Accordingly, about 10 ml urine specimen was collected from each patient in a sterile screw-capped, wide-mouthed container labelled with unique sample numbers, date, and time of collection. Immediately, they were delivered to the bacteriology laboratory and processed.

The antimicrobial susceptibility test was carried out by the Kirby–Bauer disc diffusion method as per Clinical Laboratory Standards Institute (CLSI—formerly NCCLS) guidelines on Mueller–Hinton agar (Oxoid, Basingstoke, Hampshire, England [13]). Briefly, four to five bacterial colonies were inoculated into 5 ml of Soyabean Casein Digest Broth (Oxoid, Basingstoke, Hampshire, England) and incubated at 37°C for 12 hours. After an overnight incubation, the growth suspension was prepared in 0.5 ml of the same broth medium, and the turbidity was adjusted to match that of 0.5 McFarland standards to obtain approximately the organism number of 1 × 10^6^ colony-forming units (CFU) per ml. Then, a sterile swab was dipped into this suspension, and the excess of inocula was removed by pressing it against the sides of the tube. Then, the swab was applied to the center of Mueller–Hinton agar plate (Oxoid, Basingstoke, Hampshire, England) and evenly spread on the medium. Antibiotic discs were placed after 15 minutes of inoculation to Mueller–Hinton agar seeded with each isolate and were incubated for 24 hours at 37°C. The diameter of the zone of inhibition around the disc was measured using a sliding metal caliper. Interpretation criteria were those of Clinical Laboratory Standards Institute (CLSI) guidelines. The following drugs and concentrations were used to determine the antibiogram of the strains: penicillin (10 U), augmentin (30 *μ*g), trimethoprim-sulfamethoxazole (1.25/23.75 *μ*g), clindamycin (30 *μ*g), gentamicin (30 *μ*g), ciprofloxacin (5 *μ*g), erythromycin (15 *μ*g), chloramphenicol (30 *μ*g), cefuroxime (30 *μ*g), cephalothin (10 *μ*g), ceftazidime (30 *μ*g), cefotaxim (30 *μ*g), ceftriaxone (10 *μ*g), oxacillin (1 *μ*g), nitrofurantoin (30 *μ*g), nalidixic acid (30 *μ*g), and norfloxacin (30 *μ*g). All antibiotic disks were purchased from Oxoid Limited Company, United Kingdom.

### 2.7. Quality Control Assurance

The reliability of the study findings was guaranteed by implementing quality control measures throughout the whole process of the laboratory work. Staining reagents, culture media, and antibiotic discs were checked for their normal shelf life before use. All culture plates and antibiotic discs were stored at recommended refrigeration temperature (2–8°C) after preparation. Reference strains of *S. aureus* ATCC 25923, *E. coli* ATCC 25922, and *P. aeruginosa* ATCC 27853 were used as controls. In general, all laboratory procedures were done based on recommended standard laboratory procedures by strictly following preanalytical, analytical, and postanalytical stages of quality assurance that are incorporated in standard operational procedures (SOPs) of microbiology laboratory of Yekatit 12 Hospital Medical College.

### 2.8. Data Analysis

Data were cleaned, entered, and analyzed using SPSS, version 17. Descriptive statistics was computed for most of the study variables, and frequency distribution tables were used to describe the findings. Logistical regression was also used to estimate crude odds ratio (COR) with 95% confidence interval (CI) of positive responses to the different variables, and *P* values less than 0.05 were taken as statistically significant when looking for associations between dependent and independent variables.

## 3. Results

### 3.1. Sociodemographic Characteristics of Patients

There were 384 (199 males and 185 females) pediatric patients less than or equal to 15 years, from whom urine samples were collected. Of these patients, 61 (15.9%) had significant bacteriuria. Of the 185 females, 36 (19.5%) came up with positive cultures, while 25 (12.6%) of the 199 males had significant bacteriuria, indicating that female patients were more affected than male patients. As can be seen in Table 1, the largest numbers of study subjects were below the age of 3 years, and the largest positive culture was obtained from this age group accounting for 35 (57.4%.) positive cultures out of 61 positive cultures (Table 1).

Bacterial species belonging to six genera were isolated and identified from 61 positive cultures, and the genera were *Escherichia*, *Klebsiella*, *Staphylococcus*, *Proteus*, *Acinetobacter*, and *Enterococcus*; *E. coli* was isolated from 28 cases (49.5 %), followed by *Klebsiella* spp. from 17 cases (27.9%), *Staphylococcus* spp. from 5 patients (8.2 %.), *S. aureus* from one case and coagulase-negative staphylococci from 4 cases, *Enterococcus* from 7 cases (11.5%), *Proteus* spp. from 3 cases (4.9%), and *Acinetobacter* from one case (1.6%). Of the bacterial isolates, *E. coli* was found to be the most common pathogen followed by *Klebsiella*. Furthermore, *E. coli* and *Klebsiella* were the most common pathogens in female patients accounting for 71.4% and 64.7%, respectively (Table 2).

Isolation rate of UTI with regard to circumcision revealed that out of 178 circumcised male study subjects, 10.1% (18/178) were culture-positive, but from a total of 21 uncircumcised study subjects, 33.3% (7/21) were culture-positive. Circumcision was significantly associated (COR, 95% CI: 0.225 (0.080–0.630), *P*=0.005) with urinary tract infection (Table 3).

### 3.2. Antibiotic Sensitivity Testing

The drug susceptibility profile of bacterial isolates is given in Tables 4 and 5. *E. coli* and *Klebsiella* spp. were not 100% susceptible to any of the 11 antibiotics tested. On the one hand, *Acinetobacter* spp. had 100% resistance to three antibiotics: gentamicin, trimethoprim-sulfamethoxazole, and augmentin, but they were 100% susceptible to ciprofloxacin, cefuroxime, norfloxacin, and ceftazidime. On the other hand, *Proteus* spp. were 100% sensitive to all drugs except nitrofurantoin (Table 4).

Also, a high degree of resistance was observed in *Enterococcus* spp. Species of *Enterococcus* had resistance of 71.4 to chloramphenicol and 85.7% to both trimethoprim-sulfamethoxazole and erythromycin (Table 5). As shown in Table 5, *S. aureus* was 100% susceptible to almost all drugs while coagulase-negative staphylococci were not as susceptible as *S. aureus.*

Multidrug resistance (MDR) to two or more drugs was observed in 11/12 (91.7%) and 34/49 (69.4%) of Gram-positive and Gram-negative bacteria, respectively, (Tables 6 and 7). The overall prevalence of MDR in both groups was 45/61 (73.7%).

## 4. Discussion

The present study was conducted to determine the prevalence of UTIs, to evaluate the bacterial agents involved in UTI, and to determine the drug susceptibility profile of bacterial uropathogens. A total of 384 study participants were enrolled in the present study, of whom, 199 (51.8%) were males and 185 (48.2%) were females. The ages of study subjects ranged from 0 to 15 years.

Out of the 384 study subjects (185 female and 199 males between the ages of 0 and 15 years) who participated in this study, only 61 (15.8%) had urine samples with significant bacteriuria. The findings of this study were similar to those reported in Nigeria [14], Iran [15], and Egypt [16], where prevalence rates of UTI documented were 11.96%, 16.2%, and 15.05%, respectively. However, the result obtained in this study (15.8%) appeared to be higher when compared with those reported in Philadelphia [17], USA [6], India [18], and Nigeria [19]. The prevalence rates reported were 3.3%, 7.0%, 4%, and 3.0%, respectively. On the contrary, the prevalence rate of UTI obtained in this study was much less than that reported by Olowu (1996), which was 28.1%.

The present study revealed a higher incidence of urinary tract infection in females than in males. Of the 185 females, 36 (36/185; 19.5%) came up with positive cultures, while 25 (25/199; 12.6%) of the 199 males had significant bacteriuria indicating that female patients were more affected than male patients. Furthermore, the largest numbers of study subjects were patients below the age of 3 years. Among the culture-positive study subjects, 57.4% of them were below 3 years of age. Our result in this regard was comparable to that reported in Nigeria [14]. The higher incidence of urinary tract infections in females might be a result of shorter urethra and the proximity of their reproductive organ to the anus. UTI occurs in 3% to 5% of girls and 1% of boys during childhood, with the first attack occurring in girls by 5 years, peaking during infancy and toilet training, while it is common in boys during the first year of life, especially among those who are uncircumcised because during this time of age, they get exposed to pathogens easily [15, 18].

Sixty-one bacterial isolates were recovered from this study. They were *E. coli*, *Klebsiella* spp., *Proteus* spp., *Enterococcus* spp., *Acinetobacter* spp., *S. aureus*, and coagulase-negative staphylococci. Of these, *E. coli* was the most common organism isolated from patients with significant bacteriuria and was isolated from 28 (45.9%) followed by *Klebsiella* spp. from 17 cases (27.9%), *Enterococcus* spp from 7 cases (11.5%), coagulase-negative staphylococci from 4 cases (6.6%), *Proteus* spp. from 3 cases (4.9%), and *S. aureus* from 1 case (1.6%). The pattern and the frequency of bacterial isolates obtained in this study were comparable with different study findings done in different countries. For example, a study conducted in Iran [15] showed that, among bacterial isolates, 40% accounted for *E. coli*, and this was followed by *Klebsiella* spp. (17.9%), coagulase-negative staphylococci (12.3%), *Enterococcus* spp. (8.7%), *Pseudomonas* spp. (6.7%), and *S. aureus* (3.6%). A study conducted in Nigeria [14] reported a total of 36 bacterial isolates, of which, *E. coli* was the predominant organism and was responsible for 52.77% of the cases of UTI, and this was followed by *Klebsiella* spp. (25%), *Proteus mirabilis* (13.89%), *Streptococcus faecalis* (5.56%), and *Pseudomonas aeruginosa* (2.78%). Similarly, a study conducted in Egypt [16] indicated that, among all the bacteria isolated in their study, *E. coli* and *Klebsiella* spp. were the most commonly isolated bacteria accounting for 58.1% and 41.9%, respectively This study was comparable with the study conducted in different areas [20, 24, 26].

A study conducted in Philadelphia [17] indicated uncircumcision as one of the risk factors for UTI; a study in USA [6] also showed that the prevalence of UTI was less in circumcised males (2.4%) than in uncircumcised males (20.1%). This study has also showed that circumcised (10.1%) male patients were less susceptible to bacterial pathogens than were uncircumcised (33.3%) patients.

The drug susceptibility profile of Gram-negative and Gram-positive bacteria tested in the present study was variable. For instance, *E. coli* and *Klebsiella* spp. were not 100% susceptible to any of the 11 antibiotics tested. However, percentage resistance of *Klebsiella* was much higher when compared with that of *E. coli.* Eighty-eight percent of *Klebsiella* spp. was resistant to cefotaxim, ceftazidime, trimethoprim-sulfamethoxazole, and cefuroxime. The drug susceptibility profile of *E. coli* and *Klebsiella* spp. obtained in the present study was similar with the findings in Ilorin [20]. However, drug susceptibility profile of *E. coli* in this study strongly contradicts with the findings reported in Tanzania [21]. On the one hand, *Acinetobacter* spp. had 100% resistance to four antibiotics: gentamicin, trimethoprim-sulfamethoxazole, augmentin, and nalidixic acid and 100% susceptible to ciprofloxacin, cefuroxime, norfloxacin, cefotaxim, chloramphenicol, and ceftazidime. The present finding with regard to these isolated pathogens strongly contradicted with the findings in Iran [15]. On the other hand, *Proteus* spp. was 100% sensitive to all drugs except nitrofurantoin. Also, a high degree of resistance was observed in *Enterococcus* spp. *Enterococcus* spp. were 100% sensitive to penicillin but were resistant to chloramphenicol by 71.4% and by 85.7% to both trimethoprim-sulfamethoxazole and erythromycin. Similarly, *S. aureus* was 100% susceptible to almost all drugs, while coagulase-negative staphylococci were not as susceptible as *S. aureus*. This study also showed that both Gram-positive and Gram-negative bacteria had multidrug resistance (MDR) to two or more drugs with an overall prevalence of 45/61 (73.7%) in both groups. This finding was also supported by different study findings conducted in different areas [27–30]. Finally, as a limitation, the lack of such similar studies in Ethiopia for the purposes of comparison and absence of some biochemical reagents and antibiotic discs might influence the appreciation of complete profile of isolates. Serotyping and genotyping of bacterial isolates especially multidrug-resistant organisms were not possible due to limited laboratory facility.

## 5. Conclusion and Recommendation

This study determined the prevalence of urinary tract infection in pediatric patients and highlighted the major bacterial uropathogens involved in UTI for the first time in the country. Furthermore, bacterial pathogen species and their frequency was consistent with the usually reported pattern, with *E. coli* being the most common organism isolated in cases of urinary tract infections followed by *Klebsiella* spp. Most of the bacterial isolates were multidrug resistant, and it is therefore suggested that appropriate antimicrobials should be administered to reduce the risk of multidrug-resistant organisms developing and avert ineffectiveness of antibiotics. A widespread screening program for UTI in pediatric should be implemented to know the exact prevalence and to measure resistance rate of uropathogens to commonly used antibiotics.

Finally, the authors recommended that treatments of bacterial uropathogens indicate that antibiotic selection should be based on knowledge of the local prevalence of bacterial organisms and antibiotic sensitivities rather than empirical treatment. The present study indicated that ciprofloxacin (CIP), ceftazidime (CAZ), cefotaxime (CTX), cefuroxime (CXM), clindamycin (DA), and ceftriaxone (CRO) were the best antibiotic treatment against Gram-negative and Gram-positive bacterial uropathogens, respectively, in the study area relatively.

## Figures and Tables

**Table 1:** Distribution of bacterial isolates in relation to age group among pediatric patients at Yekatit 12 Hospital Medical College, Addis Ababa, Ethiopia, 2014.

Age in years	Males with positive culture	Females with positive culture	Total	%
<3	16 (*n*=110)	19 (*n*=83)	35	57.4
3–6	1 (*n*=13)	3 (*n*=20)	4	6.6
6–9	1 (*n*=26)	8 (*n*=30)	9	14.8
9–12	2 (*n*=24)	4 (*n*=29)	6	9.8
12–15	5 (*n*=22)	2 (*n*=33)	7	11.5
Total	25 (40.9%)	36 (59.01%)	*N*=61	100

*n*: number of patients in each age group; *N*: total number of positive male and female patients in each age group.

**Table 2:** Distribution of bacterial isolates in relation to sex among pediatric patients at Yekatit 12 Hospital Medical College, Addis Ababa, Ethiopia, 2014.

Bacterial isolates	Female	Male	Total
*E. coli*	20/28 (71.4%)	8/28 (28.6)	28/61 (45.9%)
*Klebsiella* spp.	11/17 (64.7%)	6/17 (35.3)	17/61 (27.9%)
*Proteus* spp.	—	3/3 (100%)	3/61 (4.9%)
*Acinetobacter* spp.	—	1/1 (100%)	1/61 (1.6%)
*Enterococcus* spp.	4/7 (57.1)	3/7 (42.9%)	7/61 (11.5)
*S. aureus*	1/1 (100%)	—	1/61 (1.6%)
CONS	—	4/4 (100%)	4/61 (6.6%)
Total	36 (59.0)	25 (40.9%)	61 (100%)

CONS: coagulase-negative staphylococci.

**Table 3:** Association of UTI in relation to circumcision among pediatric patients at Yekatit 12 Hospital Medical College, Addis Ababa, Ethiopia, 2014 (*n*=199).

UTI				
Circumcision	No	Yes	COR 95% CI	*P* value
No	14 (66.6%)	7 (33.3%)	1	—
Yes	160 (89.9%)	18 (10.1%)	0.225 (0.080–0.630)^*∗*^	0.005

^*∗*^Significant at *P* value <0.05; COR: crude odds ratio.

**Table 4:** The number and percent of resistant strains of Gram-negative uropathogens among pediatric patients at Yekatit 12 Hospital Medical College, Addis Ababa, Ethiopia, 2014.

Bacteria isolated	Total no.	Antimicrobial agents tested										
		CIP	CXM	GN	NA	SXT	F	AMP	C	NOR	CTX	CAZ
		No. (%)	No. (%)	No. (%)	No. (%)	No. (%)	No. (%)	No. (%)	No. (%)	No. (%)	No. (%)	No. (%)
*E. coli*	28	6 (21.4)	9 (32.1)	8 (28.6)	9 (32.1)	19 (67.9)	5 (17.9)	11 (39.3)	8 (28.6)	7 (25.0)	9 (32.1)	9 (32.1)
*Klebsiella* spp.	17	5 (29.4)	15 (88.2)	14 (82.4)	9 (52.9)	15 (88.2)	5 (29.4)	12 (70.6)	10 (58.8)	6 (35.3)	15 (88.2)	15 (88.2)
*Acinetobacter* spp.	1	0	0	1 (100)	1 (100)	1 (100)	0	1 (100)	0	0	0	0
*Proteus* spp.	3	0	0	0	0	0	3 (100)	0	0	0	0	0
Total	**49**	**11 (22.5)**	**24 (48.9)**	**23 (46.9)**	**19 (38.7)**	**35 (71.4)**	**13 (26.5)**	**24 (48.9)**	**18 (36.7)**	**13 (26.5)**	**24 (48.9)**	**24 (48.9)**

AMP: augmentin (30 *μ*g); NOR: norfloxacin (30 *μ*g); GN: gentamicin (30 *μ*g); CIP: ciprofloxacin (5 *μ*g); NA: nalidixic acid (30 *μ*g); F: nitrofurantoin (30 *μ*g); CAZ: ceftazidime (30 *μ*g); CTX: cefotaxim (30 *μ*g); C: chloramphenicol (30 *μ*g); SXT: trimethoprim-sulfamethoxazole (1.25/23.75 *μ*g); CXM: cefuroxime (30 *μ*g).

**Table 5:** The number and percent of resistant strains of Gram-positive uropathogens among pediatric patients at Yekatit 12 Hospital Medical College, Addis Ababa, Ethiopia, 2014.

Bacteria isolated	Total no.	Antimicrobial agents tested												
		P	CIP	C	E	OX	CXM	GN	KF	SXT	F	DA	AMP	CRO
		No. (%)	No. (%)	No. (%)	No. (%)	No. (%)	No. (%)	No. (%)	No. (%)	No. (%)	No. (%)	No. (%)	No. (%)	No. (%)
*Enterococcus* spp.	7	0	1 (14.3)	5 (71.4)	6 (85.7)	—	3 (42.9)	0	3 (42.9)	6 (85.7)	1 (14.3)	0	3 (42.9)	3 (42.9)
CONS	4	0	1 (25.0)	1 (25.0)	4 (100)	—	4 (100)	0	0	4 (100)	0	0	1 (25.0)	1 (25.0)
*S. aureus*	1	1 (100)	0	0	1 (100)	0	0	0	1 (100)	0	0	0	1 (100)	0
Total	**12**	**1 (8.3)**	**2 (16.6)**	**6 (50)**	**11 (91.6)**	**0**	**7 (58.3)**	**0**	**4 (33.3)**	**10 (83.3)**	**1 (8.3)**	**0**	**5 (41.6)**	**4 (33.3)**

CONS: coagulase-negative staphylococci; P: penicillin (10 U); AMP: augmentin (30 *μ*g); KF: cephalothin (10 *μ*g); DA: clindamycin (30 *μ*g); CRO: ceftriaxone (10 *μ*g); GN: gentamicin (30 *μ*g); E: erythromycin (15 *μ*g); CIP: ciprofloxacin (5 *μ*g); OX: oxacillin (1 *μ*g); F: nitrofurantoin (30 *μ*g); C: chloramphenicol (30 *μ*g); SXT: trimethoprim-sulfamethoxazole (1.25/23.75 *μ*g); CXM: cefuroxime (30 *μ*g).

**Table 6:** Multidrug resistance pattern of Gram-negative bacteria isolated from pediatric patients at Yekatit 12 Hospital Medical College, Addis Ababa, Ethiopia, 2014.

Combination of antibacterial agent	*E. coli* no. (%)	*Klebsiella* spp. no. (%)	*Acinetobacter* spp. no. (%)	Total no. (%)
AMP, SXT	3 (16.6)	—	—	3 (8.8)
C, SXT	3 (16.6)	—	—	3 (8.8)
CIP, NOR, SXT	1 (5.6)	—	—	1 (2.94)
AMP, SXT, NA, GN	1 (5.6)	—	1 (100)	2 (5.9)
AMP, CIP, SXT, NOR, GN, CAZ, CTX, CXM	4 (22.2)	—	—	4 (11.8)
AMP, SXT, NA, GN, CAZ, CTX, CXM, C	4 (22.2)	4 (26.7)	—	8 (23.5)
CIP, SXT, NA, GN, CAZ, CTX, CXM, NOR, F	1 (5.6)	5 (33.3)	—	6 (17.6)
AMP, SXT, NA, GN, CAZ, CTX, CXM, NOR, F	1 (5.6)	6 (40)	—	7 (20.6)
Total	**18 (100)**	**15 (100)**	**1 (100)**	**34 (100)**

AMP: augmentin (30 *μ*g); NOR: norfloxacin (30 *μ*g); GN: gentamicin (30 *μ*g); CIP: ciprofloxacin (5 *μ*g); NA: nalidixic acid (30 *μ*g); F: nitrofurantoin (30 *μ*g); CAZ: ceftazidime (30 *μ*g); CTX: cefotaxim (30 *μ*g); C: chloramphenicol (30 *μ*g); SXT: trimethoprim-sulfamethoxazole (1.25/23.75 *μ*g); CXM: cefuroxime (30 *μ*g).

**Table 7:** Multidrug resistance pattern of Gram-positive bacteria isolated from pediatric patients at Yekatit 12 Hospital Medical College, Addis Ababa, Ethiopia, 2014.

Combination of antibacterial agent	*Enterococcus* spp. no. (%)	*S. aureus* no. (%)	CONS no. (%)	Total no. (%)
P, AMP, KF	—	1 (100)	—	1 (9.09)
P, SXT, E, CXM	—	—	3 (75)	3 (27.2)
P, SXT, E, C	2 (33.3)	—	—	2 (18.2)
P, SXT, E, C, AMP	1 (16.7)	—	1 (25)	2 (18.2)
CXM, E, F, AMP, SXT	1 (16.7)	—	—	1 (9.09)
CXM, E, F, KF, CRO, SXT, C	1 (16.7)	—	—	1 (9.09)
C, E, F, KF, CRO, SXT	1 (16.7)	—	—	1 (9.09)
Total	6 (100)	1 (100)	4 (100)	11 (100)

CONS: coagulase-negative staphylococci; P: penicillin (10 U); AMP: augmentin (30 *μ*g); KF: cephalothin (10 *μ*g); DA: clindamycin (30 *μ*g); CRO: ceftriaxone (10 *μ*g); GN: gentamicin (30 *μ*g); E: erythromycin (15 *μ*g); CIP: ciprofloxacin (5 *μ*g); OX: oxacillin (1 *μ*g); F: nitrofurantoin (30 *μ*g); C: chloramphenicol (30 *μ*g); SXT: trimethoprim-sulfamethoxazole (1.25/23.75 *μ*g); CXM: cefuroxime (30 *μ*g).

## Data Availability

The data used to support the findings of this study cannot be shared in a publicly available data repository system, because there is no such a data repository system in the country. However, the data are available form the authors upon request.

## Abbreviations

AST: Antimicrobial susceptibility testing
BA: Blood agar
CLED: Cystine-lactose-electrolyte deficient
CI: Confidence interval
CONS: Coagulase-negative staphylococci
CLSI: Clinical Laboratory Standards Institute
DMLT: Department of Medical Laboratory Technology
ESBL: Extended-spectrum *β*-lactamase
KIA: Kligler iron agar
IRB: Institutional Review Board
MSU: Midstream urine samples
NCCLS: National Committee for Clinical Laboratory Standards
OPD: Outpatient Department
QC: Quality control
SOPs: Standard operating procedures
Spp: Species
SPSS: Statistical Package for Social Sciences
UTI: Urinary tract infection
USA: United States of America
WHO: World Health Organization.
